# Computed Tomography of the Nasal Cavities and Paranasal Sinuses of Cattle at Different Ages

**DOI:** 10.1002/vms3.70893

**Published:** 2026-03-31

**Authors:** Charles Montel, Florian Isler, Benjamin Cartiaux, Renaud Maillard, Giovanni Mogicato

**Affiliations:** ^1^ ToNIC, Toulouse NeuroImaging Center, Université de Toulouse, INSERM, UPS, Ecole Nationale Veterinaire de Toulouse Toulouse Occitanie France; ^2^ Clinique vétérinaire Mon Véto, Bourg‐Achard, Ecole Nationale Veterinaire de Toulouse Toulouse Occitanie France; ^3^ IHAP, Université de Toulouse, INRAE, Ecole Nationale Veterinaire de Toulouse Toulouse Occitanie France

**Keywords:** cattle, computed tomography, nasal cavities, paranasal sinuses

## Abstract

**Background:**

The nasal cavities and paranasal sinuses of cattle are complex anatomical structures that undergo significant changes throughout growth and maturation. Computed tomography (CT) is increasingly used as a routine imaging modality in small animals but remains less common in cattle due to technical limitations. However, it provides valuable diagnostic insight into the nasal cavities and sinuses, which are difficult to assess and often involved in disease processes.

**Objectives:**

This study aimed to provide a detailed description of these structures using CT in cattle of different ages in order to provide a valuable tool to clinicians.

**Methods:**

CT bone window scans of thirteen bovine heads aged 5 days to 5 years and belonging to two breeds, Holstein and Blonde d'Aquitaine, were obtained in ventral recumbency. CT and gross anatomical data were correlated with CT images and cross‐sectional anatomy from the head of three 5‐year‐old cows. Relevant anatomical structures were identified and labelled on each CT image and anatomical cross section in three different planes.

**Results:**

CT scans produced an excellent definition of the bony and air‐filled structures and allowed us to successfully identify most of the bovine head bone structures. The results revealed a progressive pneumatization of the paranasal sinuses with age, as well as an increased ossification and complexity of the nasal turbinates. Age‐related differences were particularly evident in the extent and shape of the frontal sinus.

**Conclusions:**

This study highlights the importance of considering age when interpreting CT images of the bovine head, especially in clinical and research contexts. The findings provide a reference for normal anatomical variation across different life stages and contribute to improved diagnostic accuracy in bovine veterinary imaging.

## Graphical abstract

Computed tomography (CT) provides a detailed, non‐invasive evaluation of the nasal cavities and paranasal sinuses in cattle. Age‐related changes include progressive enlargement of the sinuses and increasing ossification of the nasal turbinates. These findings highlight the importance of considering age when interpreting CT images in bovine diagnostics and anatomy.

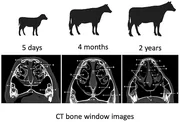

## 1 Introduction

In cattle, as in other domestic mammals, nasal cavities occupy a large part of the face and extend caudally up to the transverse bony septum at the rostral end of the cranial cavity (Singh 2017; Barone 1997). The conchae, which are coiled laminae that intrude on these cavities, comprise a caudal ethmoidal system and a rostral system in which large dorsal and ventral conchae predominate. Paranasal sinuses are bilateral air‐filled cavities in the skull that form by evagination of the nasal mucosa into the spongy bone between the external and internal plates of cranium bones. The complete set of sinuses is very complicated in cattle and constantly changes throughout life (Turgut et al. 2023, 2024).

These anatomical structures are clinically significant, as they may be affected by various diseases, including infections, neoplasia, trauma and dental‐related conditions (Angelo et al. 2009; Conti Díaz et al. 2003; Larsen 2000; Nandi et al. 2009; Russell et al. 2009). These diseases can affect cattle from a young age until adulthood (Braun et al. 2015; Narita et al. 1982; Njoku and Chineme 1983; Ward and Rebhun 1992). However, the deep location of paranasal sinuses and bony encapsulation make clinical exploration particularly challenging in bovine species (Anderson and St Jean 2008; Schleining 2016). Although conventional radiography is frequently used to examine the nasal cavity and sinuses, its utility is limited due to the superimposition of surrounding anatomical features. In contrast, computed tomography (CT) offers detailed cross‐sectional imaging and has been shown to be more effective for identifying, localizing, extending and characterizing lesions of the nasal cavities (Seeram 2010; Stanitznig et al. 2018). For example, CT can provide valuable diagnostic information in cases of bacterial sinusitis, traumatic injuries, congenital malformations or suspected space‐occupying lesions, as it allows precise evaluation of the bony structures and the extent of soft‐tissue involvement (Turgut et al. 2023).

Although CT is commonly used in carnivores (De Rycke et al. 2003; Losonsky et al. 1997) and equines (Goodarzi et al. 2021) for diagnostic purposes and anatomical studies, it remains underutilized in bovine practice. This underuse is largely due to the logistical difficulties of imaging large animals. Nevertheless, recent studies have applied CT to analyse the anatomy of the paranasal sinuses in adult Holstein cows (Turgut et al. 2023), pigs (Kyllar et al. 2014), camels (Ben Khalifa et al. 2019; Kabkia et al. 2025), small ruminants (Alsafy et al. 2021; Awaad et al. 2019; Masoudifard et al. 2022; Tohidifar et al. 2020), buffaloes (Alsafy et al. 2013), Zebu cattle (Nomir et al. 2024) and primates such as lemurs (Mogicato et al. 2012) and New World monkeys (Smith et al. 2024).

However, unlike previous CT‐based studies that focused exclusively on adult cattle, the present work offers a developmental perspective by documenting progressive morphological changes in the nasal cavities and paranasal sinuses from a young age until adulthood. This age‐based comparative approach provides new anatomical reference data that is directly relevant to clinicians interpreting CT scans in young or growing animals.

Thus, the objective of this study was to analyse normal bovine head anatomy using CT imaging in animals of various ages, with a focus on describing the nasal cavities and paranasal sinuses. We aimed to generate age‐specific anatomical reference data to aid clinicians in diagnostic and surgical applications, as well as educational purposes.

## 2 Materials and Methods

### 2.1 Animals

Thirteen bovine heads from two breeds (Holstein and Blonde d'Aquitaine), ranging in age from 5 days to 5 years, were collected following euthanasia and submitted for necropsy at Ecole Nationale Vétérinaire de Toulouse (France).

All animals were euthanized for reasons unrelated to this study, following standard veterinary procedures using intravenous administration of pentobarbital sodium (Dolethal, Vetoquinol). Post‐mortem sample collection and experimental protocol were approved by the ethical committee Sciences et Santé Animale 115 ‐ Ecole Nationale Vétérinaire de Toulouse (France), and informed owner consent was obtained. The heads were washed with tap water and prepared for further processing.

### 2.2 Computer Tomographic Imaging

Within 24 h post‐mortem, CT scans of all heads were performed using a 16‐slice helical CT scanner (Optima CT540, GE Healthcare, USA). The heads were positioned with the mandible resting on the CT table and aligned perpendicularly to the hard palate in the rostrocaudal direction. Dorsoventral and lateral scout views ensured correct alignment. Scanning was conducted from the nasal planum to the atlanto‐occipital joint. Images were acquired using a 0.625 cm slice thickness at 1 cm intervals. Both soft tissue and bone reconstruction algorithms were applied, and the images were archived in DICOM format.

### 2.3 CT and Gross Anatomical Correlation

To correlate CT images with anatomical structures, the head of three 5‐year‐old cows was scanned and then frozen at −20°C for 24 h and sectioned transversely and sagittally into 6 cm thick slices using a band saw. The slices were cleaned gently by light brushing under tap water. The section surface of each slice was photographed using a digital camera with the caudal surface of each slice facing the camera for transverse sections and with the lateral surface for sagittal sections.

Bone window (CT image reconstruction setting optimized for bone detail) (window level 300 Hounsfield unit [HU] and window width 2800 HU) and soft tissue window (CT image reconstruction setting optimized for soft tissue detail) (window level 50 HU and window width 500 HU) images were reviewed for nasal and sinonasal diseases, anomalies and assessment of image quality. DICOM CT data were opened in a viewer software (Horos, Horos Project, USA). Premolar and molar teeth were used as landmarks to explore and describe the location and exploration of the structures and cavities. The anatomical structures were labelled using image processing software (PowerPoint, Microsoft, USA). Letters are used to label the bones, whereas numbers indicate the other anatomical structures of the nasal cavities. Structures were named according to anatomy texts and the *Nomina Anatomica Veterinaria* (World Association of Veterinary Anatomists 2017).

Due to the small sample size and breed representation, relative sinus size descriptions were based on visual comparison of CT images in bone window settings across planes, rather than quantitative volumetric measurements.

## 3 Results

CT analysis of the 13 individuals studied, belonging to the Prim'Holstein and Blonde d'Aquitaine breeds and aged from 5 days to 5 years, made it possible to characterize the morphological evolution of the nasal cavities and paranasal sinuses during growth.

CT imaging allowed detailed assessment of sinonasal anatomy and developmental changes across age groups. An atlas comprising 40 CT bone window images from 13 animals was created, including eight transverse, one dorsal and one sagittal (paramedian) plane. Four animals aged 5 days, 4 months, 2 years and 5 years were selected for representative depiction.

**FIGURE 1:**
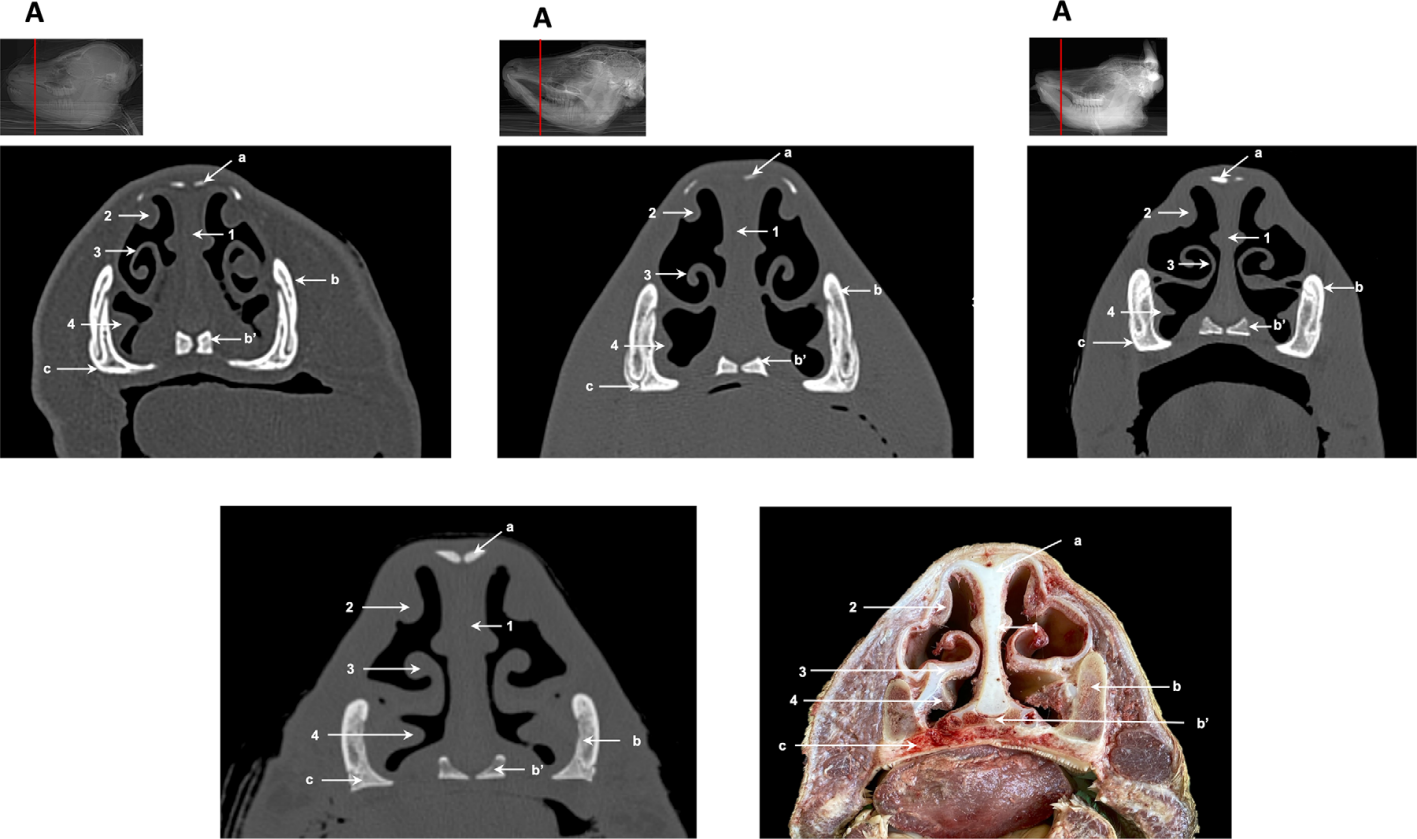
Transverse planes corresponding to reference Line A of the scout image. Images above: from left to right, computed tomography images from animals aged, respectively, 5 days, 4 months and 2 years. Images below: corresponding computed tomography and cross‐sectional anatomy images from animal aged 5 years. 1. Nasal septum. 2. Straight fold. 3. Alar fold. 4. Basal fold. a. Nasal bone. b. Nasal process of the incisive. b′. Palatine process of the incisive bone. c. Maxilla.

**FIGURE 2:**
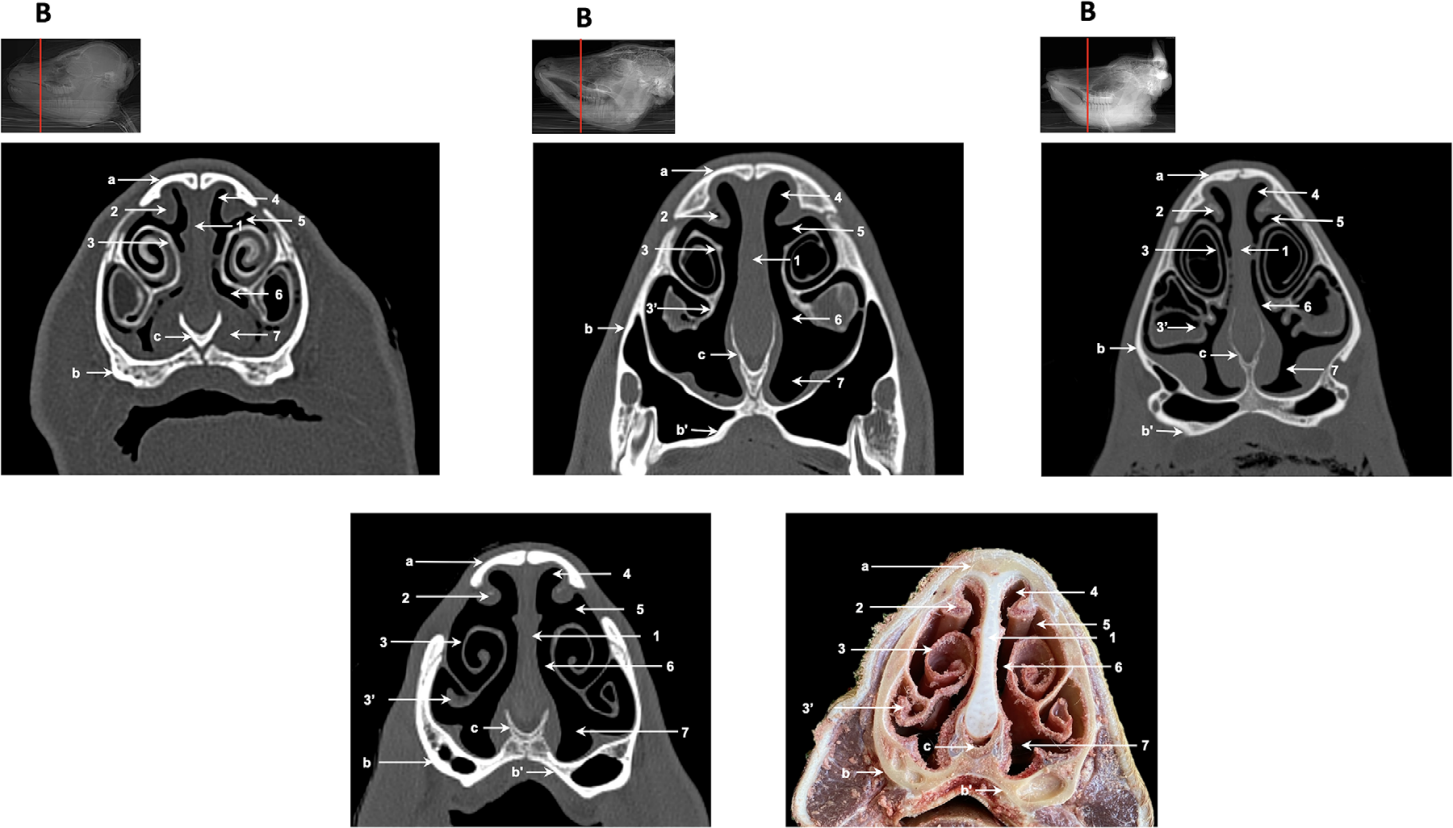
Transverse planes corresponding to reference Line B of the scout image. Images above: from left to right, computed tomography images from animals aged, respectively, 5 days, 4 months and 2 years. Images below: corresponding computed tomography and cross‐sectional anatomy images from animal aged 5 years. 1. Nasal septum. 2. Dorsal nasal concha. 3. Dorsal spiral lamellae of the ventral nasal concha. 3′. Ventral spiral lamellae of the ventral nasal concha. 4. Dorsal nasal meatus. 5. Middle nasal meatus. 6. Common nasal meatus. 7. Ventral nasal meatus. a. Nasal bone. b. Maxilla. b′. Palatine process of maxilla. c. Vomer.

**FIGURE 3:**
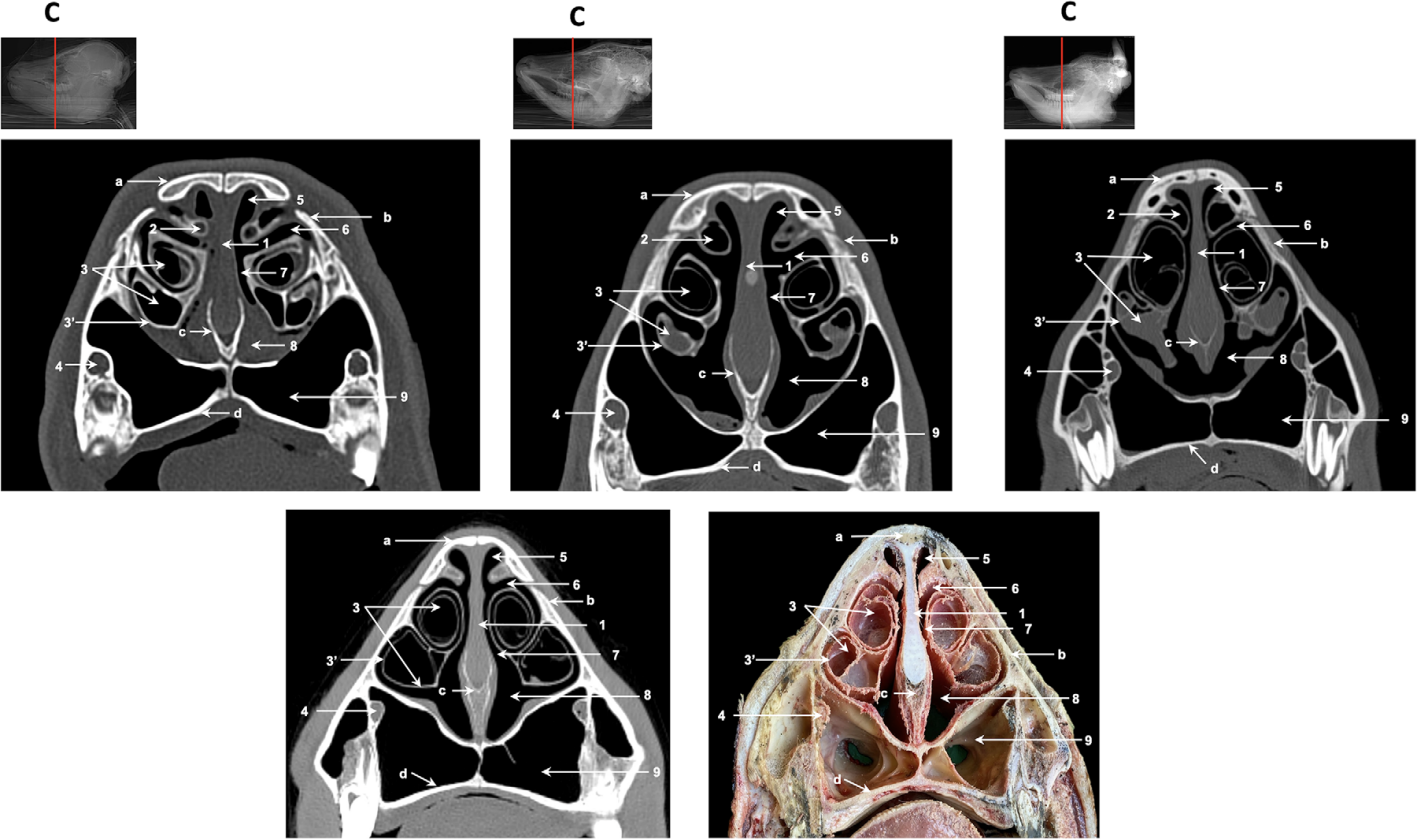
Transverse planes corresponding to reference Line C of the scout image. Images above: from left to right, computed tomography images from animals aged, respectively, 5 days, 4 months and 2 years. Images below: corresponding computed tomography and cross‐sectional anatomy images from animal aged 5 years. 1. Nasal septum. 2. Dorsal conchal sinus. 3. Ventral nasal concha. 3′. Ventral spiral lamellae of the ventral nasal concha. 4. Infraorbital canal. 5. Dorsal nasal meatus. 6. Middle nasal meatus. 7. Common nasal meatus. 8. Ventral nasal meatus. 9. Palatine sinus. a. Nasal bone. b. Lacrimal bone. c. Vomer. d. Palatine process of maxilla.

**FIGURE 4:**
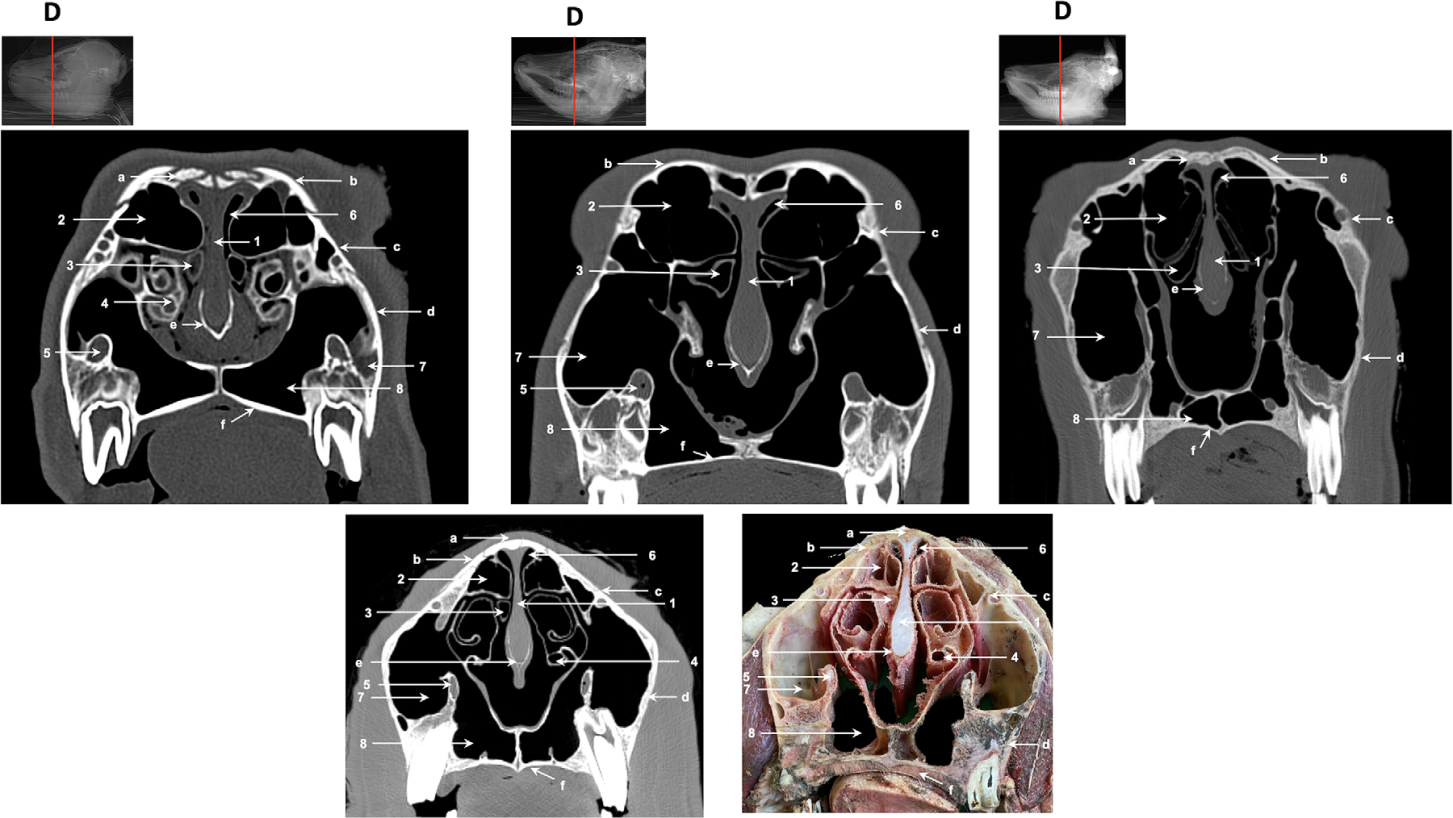
Transverse planes corresponding to reference Line D of the scout image. Images above: from left to right, computed tomography images from animals aged, respectively, 5 days, 4 months and 2 years. Images below: corresponding computed tomography and cross‐sectional anatomy images from animal aged 5 years. 1. Nasal septum. 2. Dorsal conchal sinus. 3. Middle conchal sinus. 4. Ventral conchal sinus. 5. Infraorbital canal. 6. Dorsal nasal meatus. 7. Maxillary sinus. 8. Palatine sinus. a. Nasal bone. b. Frontal bone. c. Lacrimal bone. d. Maxilla. e. Vomer. f. Palatine bone.

**FIGURE 5:**
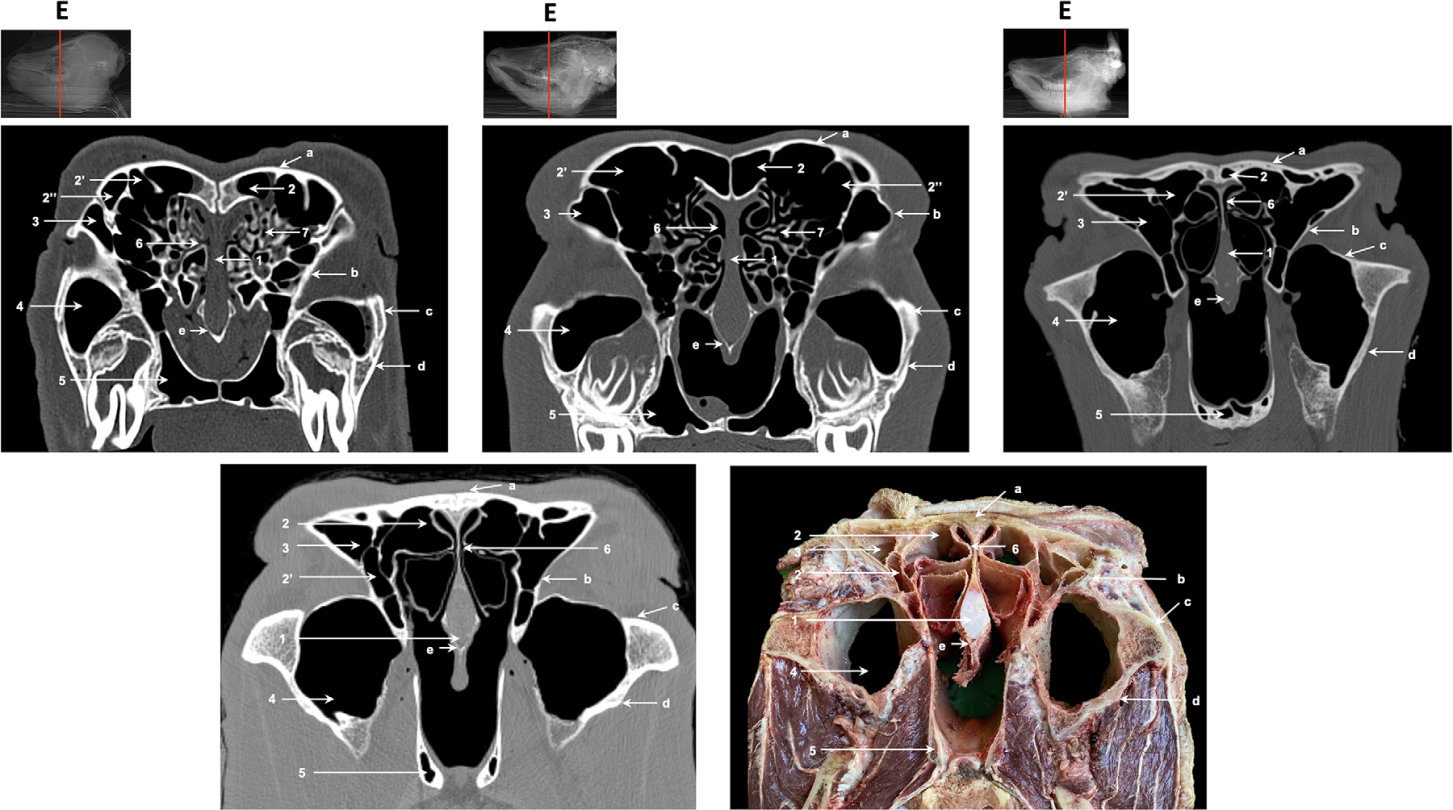
Transverse planes corresponding to reference Line E of the scout image. Images above: from left to right, computed tomography images from animals aged, respectively, 5 days, 4 months and 2 years. Images below: corresponding computed tomography and cross‐sectional anatomy images from animal aged 5 years. 1. Nasal septum. 2. Medial rostral frontal sinus. 2′. Intermediate rostral frontal sinus. 2″. Lateral rostral frontal sinus. 3. Lacrimal sinus. 4. Maxillary sinus. 5. Palatine sinus. 6. Common nasal meatus. 7. Ethmoidal labyrinth. a. Frontal bone. b. Lacrimal bone. c. Zygomatic bone. d. Maxilla. e. Vomer. f. Palatine bone.

**FIGURE 6:**
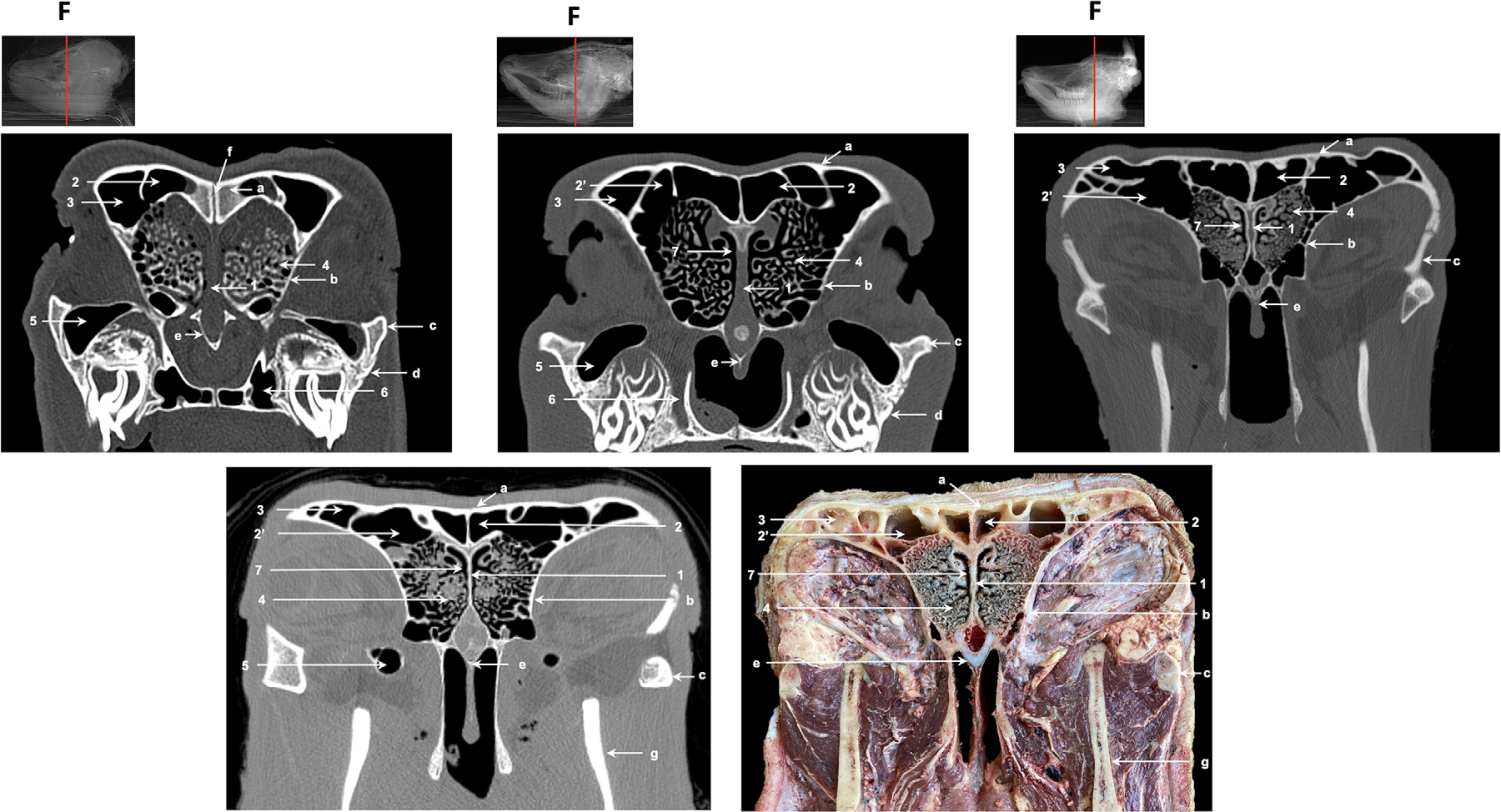
Transverse planes corresponding to reference Line F of the scout image. Images above: from left to right, computed tomography images from animals aged, respectively, 5 days, 4 months and 2 years. Images below: corresponding computed tomography and cross‐sectional anatomy images from animal aged 5 years. 1. Nasal septum. 2. Medial rostral frontal sinus. 2′. Intermediate rostral frontal sinus. 3. Rostral compartment of caudal frontal sinus. 4. Ethmoidal labyrinth. 5. Maxillary sinus. 6. Palatine sinus. 7. Common nasal meatus. a. Frontal bone. b. Lacrimal bone. c. Zygomatic bone. d. Maxilla. e. Vomer. f. Interfrontal septum. g. Mandible.

**FIGURE 7:**
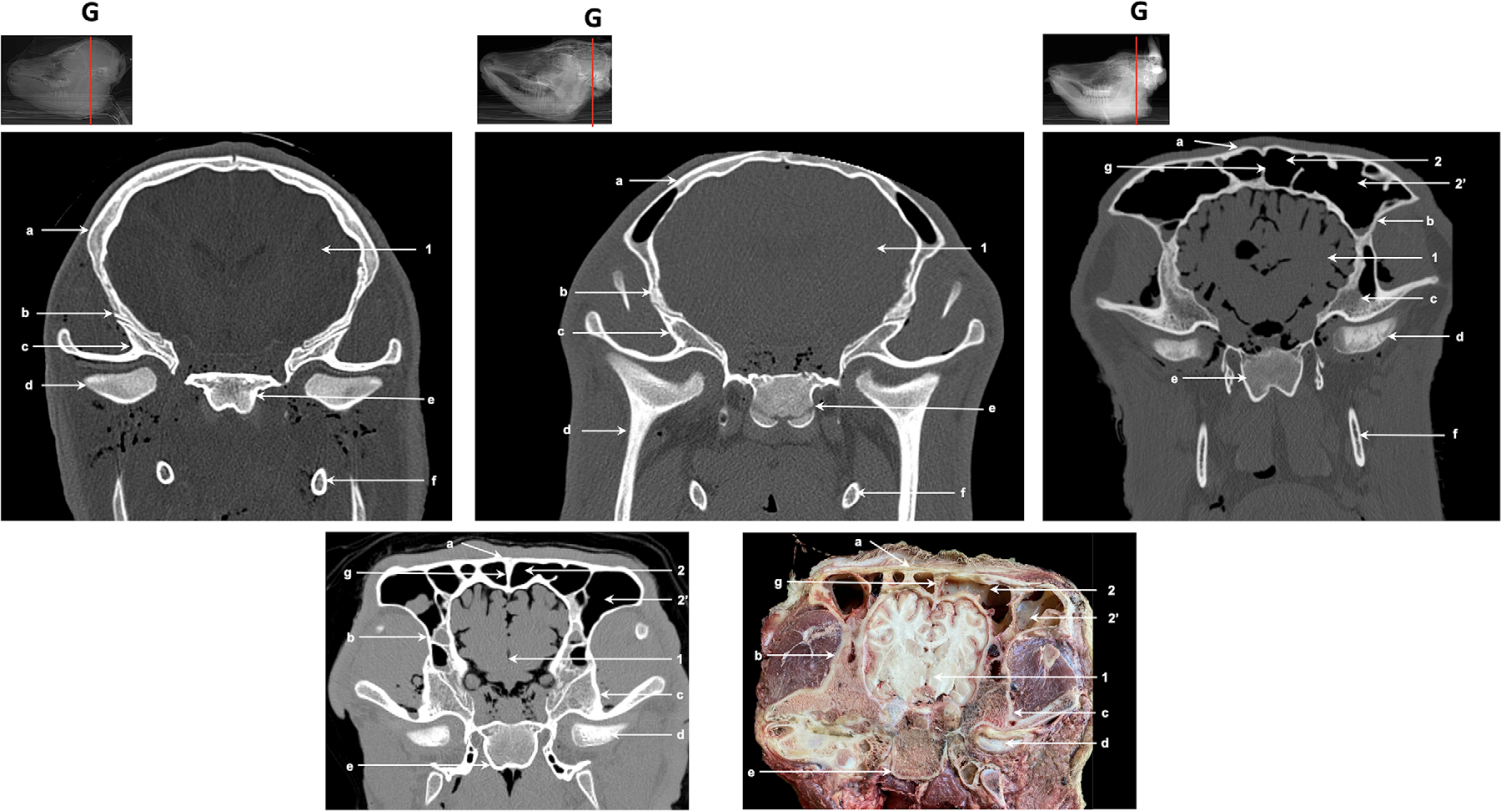
Transverse planes corresponding to reference Line G of the scout image. Images above: from left to right, computed tomography images from animals aged, respectively, 5 days, 4 months and 2 years. Images below: corresponding computed tomography and cross‐sectional anatomy images from animal aged 5 years. 1. Brain. 2. Rostral compartment of caudal frontal sinus. 2′. Intermediate compartment of caudal frontal sinus. a. Frontal bone. b. Parietal bone. c. Temporal bone. d. Mandible. e. Basisphenoid bone. f. Stylohyoid bone. g. Interfrontal septum.

**FIGURE 8:**
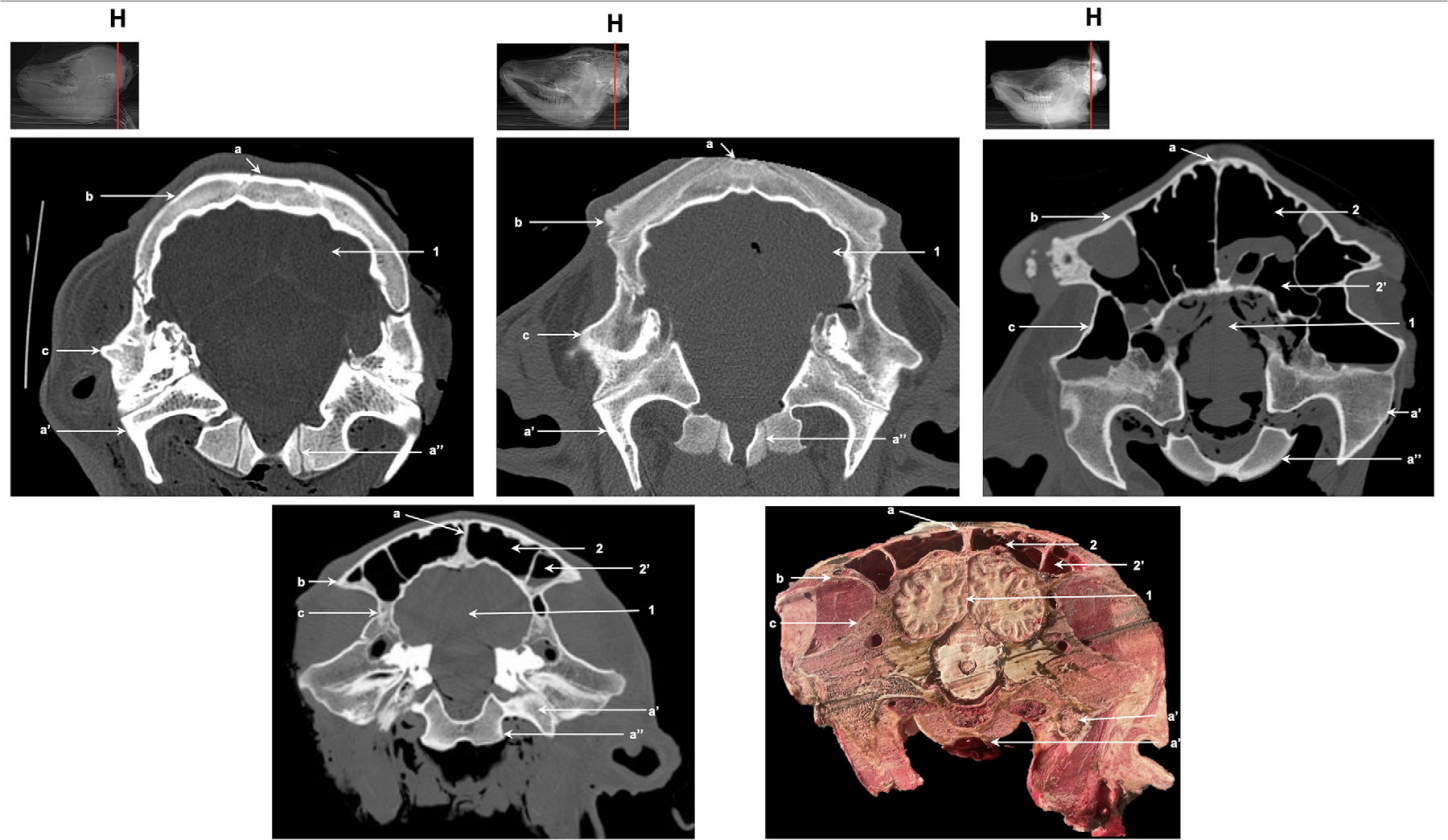
Transverse planes corresponding to reference Line H of the scout image. Images above: from left to right, computed tomography images from animals aged, respectively, 5 days, 4 months and 2 years. Images below: corresponding computed tomography and cross‐sectional anatomy images from animal aged 5 years. 1. Brain. 2. Intermediate compartment of caudal frontal sinus. 2′. Nuchal compartment of caudal frontal sinus. a. Occipital bone. a′. Jugular process of the occipital bone. a″. Basilar part of the occipital bone. b. Parietal bone. c. Temporal bone. d. Mandible. e. Basisphenoid bone. f. Stylohyoid bone.

**FIGURE 9:**
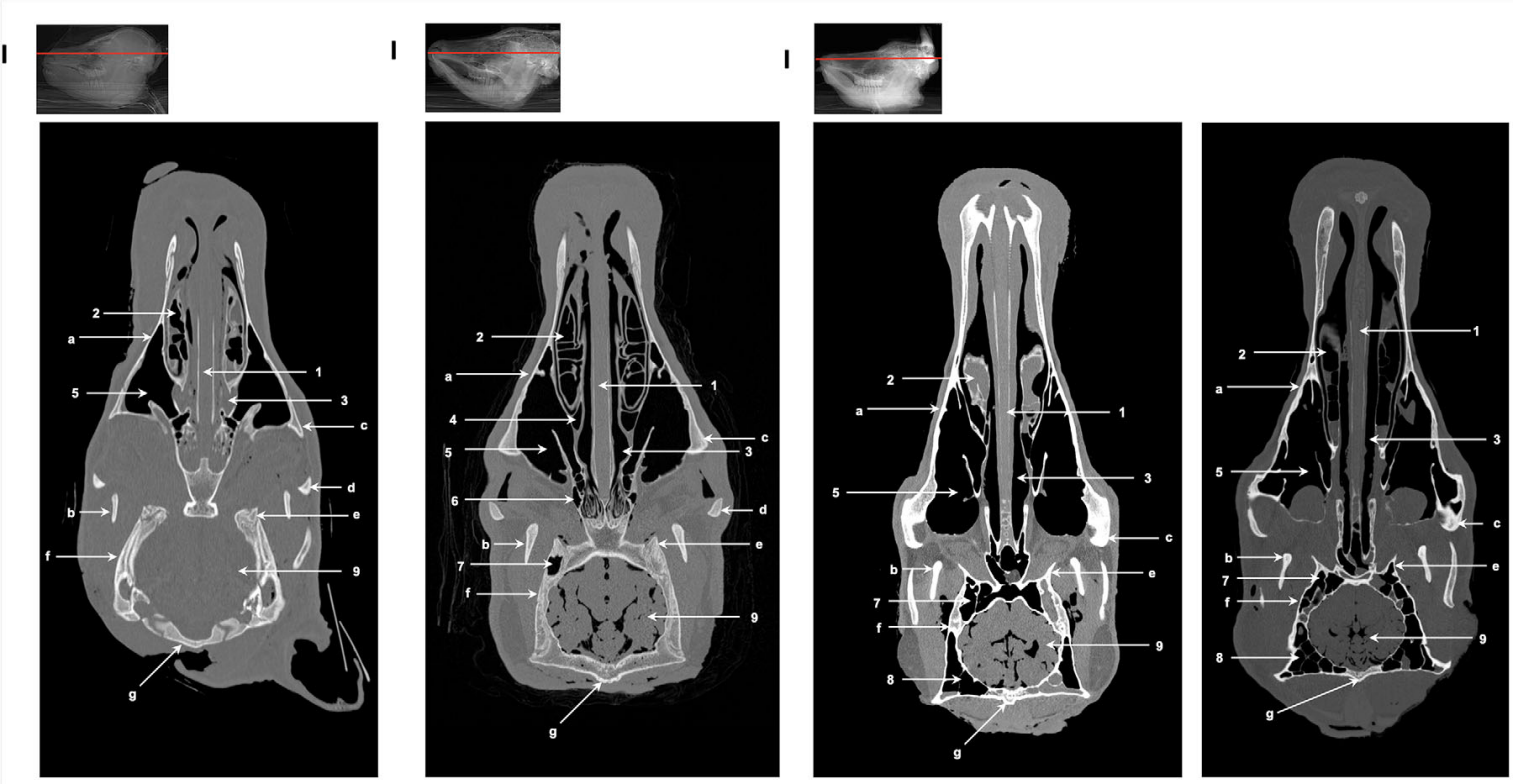
Dorsal planes corresponding to reference Line I of the scout image. From left to right, computed tomography images from animals aged, respectively, 5 days, 4 months, 2 years and 5 years. 1. Nasal septum. 2. Ventral nasal concha. 3. Middle nasal concha. 4. Ventral conchal sinus. 5. Maxillary sinus. 6. Palatine sinus. 7. Sphenoidal sinus. 8. Caudal frontal sinus. 9. Brain. a. Maxilla. b. Coronoid process of the mandible. c. Zygomatic bone. d. Frontal process of the zygomatic bone. e. Basisphenoid bone. f. Temporal bone. g. Occipital bone.

**FIGURE 10:**
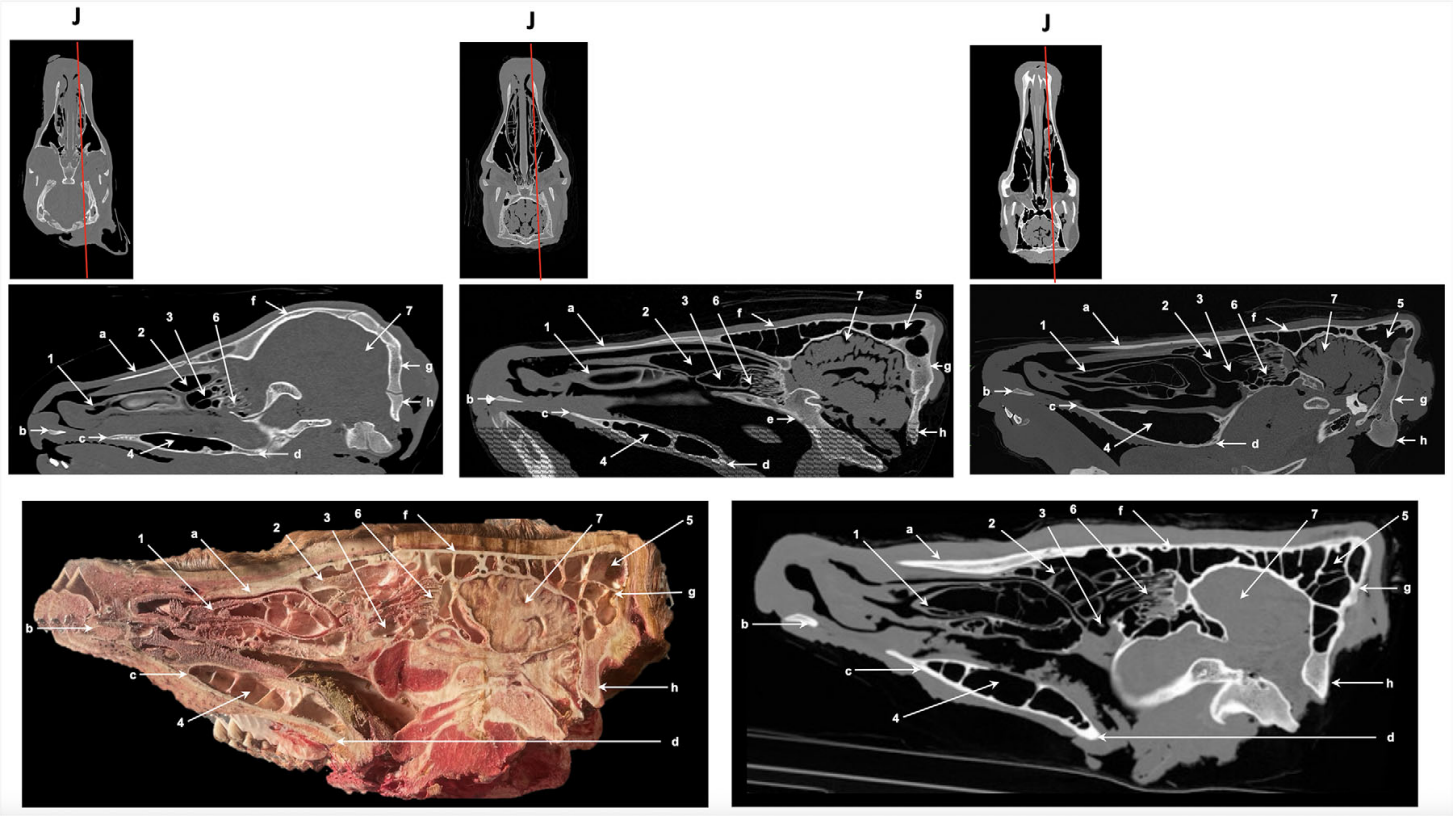
Sagittal (paramedian) planes corresponding to reference Line J of the dorsal image. Images above: from left to right, computed tomography images from animals aged, respectively, 5 days, 4 months and 2 years. Images below: corresponding computed tomography and sagittal anatomy images from animal aged 5 years. 1. Ventral nasal concha. 2. Dorsal conchal sinus. 3. Middle conchal sinus. 4. Palatine sinus. 5. Caudal frontal sinus. 6. Ethmoidal labyrinth. 7. Brain. a. Nasal bone. b. Coronoid process of the mandible. c. Palatine process of the maxilla. d. Palatine bone. e. Basisphenoid bone. f. Frontal bone. g. Parietal bone. h. Occipital bone.

The transverse CT images were chosen so that any morphological variations in the principal structures could be followed throughout the nasal cavities and corresponded to reference Lines A to H of the scout image (a preliminary CT topogram used for slice positioning). They are represented in a rostral‐to‐caudal progression from the level of the interdental space to the level of the nuchal line (Figures 1, 2, 3, 4, 5, 6, 7, 8). The dorsal CT image corresponded to reference Line I of the scout image (Figure 9). Finally, the sagittal (paramedian) CT image corresponded to reference Line J of the dorsal view (Figure 10).

### 3.1 Nasal Cavities

The nasal cavities of the cow are paired structures separated medially by the osseocartilaginous nasal septum, which forms the medial wall of each cavity (Figures 1, 2, 3, 4, 5, 6 and 9). Laterally, the cavities are bounded by the maxilla and the ethmoturbinates, forming a highly convoluted lateral wall. The dorsal wall (roof) is composed primarily of the nasal, frontal and ethmoid bones, whereas the ventral wall (floor) corresponds to the hard palate, separating the nasal cavity from the oral cavity. Within each nasal cavity, several scroll‐like nasal conchae (dorsal, ventral and ethmoidal) subdivide the space into distinct passages: the dorsal, middle, ventral and common nasal meatuses. These meatuses serve to guide inspired air toward the olfactory and respiratory regions (Figures 1, 2, 3, 4, 5, 6, 9 and 10). Age‐related development includes enlargement of conchae and increased complexity of ethmoturbinates. At 5 days, the turbinates are rudimentary, with low scroll density (Figures 9 and 10). By 4 months, the turbinates become more complex with defined folding, and at 2 years, the nasal passages are significantly larger, and the turbinates are ossified and highly structured, contributing to effective air filtration and olfaction (Figure 10).

### 3.2 Paranasal Sinuses

CT imaging revealed symmetrical sinuses without abnormalities. CT datasets were evaluated along with several adjacent structures, such as teeth, nasopharynx and soft palate, using which the paranasal sinuses and adjacent anatomical structures in cows were described in as much detail as possible. The different facial bones forming the outer wall of the nasal conchae, septa, meatuses and all ethmoturbinates were easily identified (Figures 1, 2, 3, 4, 5, 6, 7, 8, 9, 10).

The paranasal sinuses of cows on both sides of the head were composed of the dorsal conchal sinus, middle conchal sinus, ventral conchal sinus, maxillary sinus, lacrimal sinus, palatine sinus, frontal sinus, sphenoidal sinus and ethmoidal cells.

#### 3.2.1 Conchal Sinuses

The dorsal conchal sinus was present caudally in the dorsal concha (Figures 3 and 4). The middle and ventral conchal sinuses were variably present, with the ventral conchal sinus noted only in the oldest animal. Their development is progressive and correlates with nasal cavity enlargement. The middle conchal sinus was located in the middle nasal concha and extended ventral to the dorsal conchal sinus rostrally from the cribriform plate (Figure 4). The ventral conchal sinus was observed in the caudal part of the ventral nasal concha but only in 5‐year‐old cows. It was asymmetrical in structure and size (Figure 4).

#### 3.2.2 Maxillary Sinus (Figures 4, 5, 6 and 9)

Located in the maxilla and lacrimal bones, the maxillary sinus was triangular and connected to the middle nasal meatus via the nasomaxillary opening and to the palatine sinus via the maxillopalatine opening. About 2–3 cm ventral to the orbit, the sinus continued caudally into the thin‐walled lacrimal bulla and zygomatic bone. In young calves, it was small and shallow but expanded markedly by 2 years.

#### 3.2.3 Lacrimal Sinus (Figure 5 (number 3))

The lacrimal sinus was a small cavity in the lacrimal bone rostromedial to the orbit, connected to the maxillary sinus. It remained relatively small across all age groups.

#### 3.2.4 Palatine Sinus

Situated in the palatine and maxillary bones, this sinus was larger than the maxillary sinus and subdivided by an incomplete bony crest (Figure 4 (number 7)/Figure 5 (number 5)/Figure 6 (number 6)/Figure 9 (number 6)). The caudal part of the sinus was traversed obliquely by the infraorbital canal that was divided it into medial and lateral compartments. It connected with the maxillary sinus dorsally. Ossification and cavity definition improved with age.

#### 3.2.5 Frontal Sinuses (Figures 5, 6, 7, 8, 9, 10)

The frontal sinuses were the most extensive sinuses, subdivided into several compartments, within the frontal and parietal bones rostrodorsal to the brain and extended well into the cornual process of the frontal bone. The sinus cavity appeared irregular rectangular in shape and was subdivided into numerous cavities by numerous partitions that were highly variable in sizes and positions between individuals. At 5 days, the sinus was barely visible; by 2 years, it was highly compartmentalized.

#### 3.2.6 Sphenoidal Sinus (Figure 9)

The sphenoid sinus, small and asymmetrical, was located in the sphenoid bone and communicated with the ethmoidal meatus. It was noticeable small and shallow only in three specimens. It was only clearly visible in animals older than 4 months.

#### 3.2.7 Ethmoidal Cells (Figures 5, 6 and 10)

The ethmoidal cells were situated in the ethmoid bone. These honeycomb‐like air spaces communicated with the ethmoidal meatus. They were consistently present and expanded gradually with age.

### 3.3 Developmental Changes

CT findings revealed that the nasal cavity increased in volume and structural complexity with age. In neonatal calves, the conchae were underdeveloped, whereas in adults, the nasal turbinates were well‐defined and densely packed, optimizing airflow regulation.

The paranasal sinuses developed non‐linearly. Maxillary sinuses appeared early but expanded significantly after 4 months. Frontal sinuses were poorly developed at birth but showed substantial volumetric and structural growth between 4 months and 2 years. Sphenoidal and ethmoidal regions showed more gradual and delayed development, with ossification of surrounding bones becoming more prominent after 6 months.

### 3.4 CT and Gross Anatomical Correlation

Comparative analysis between CT and gross anatomical sections demonstrated excellent correspondence of bone and sinus boundaries. For instance, the maxillary sinus, nasomaxillary opening and dorsal conchal sinus observed on transverse CT images corresponded precisely to those visible on anatomical slices. Similarly, the ethmoturbinates and palatine sinus contours matched between modalities, confirming CT accuracy in depicting sinonasal structures.

## 4 Discussion

The detailed anatomical exploration of the bovine nasal cavity and paranasal sinuses presented in this study enriches our understanding of these complex craniofacial structures and provides comparative perspectives across species. Our results show a marked evolution of the nasal cavities and paranasal sinuses with age, characterized by an increase in volume and structural complexity. The use of CT thus makes it possible to precisely document these modifications, providing a dynamic view of sinonasal development in domestic cattle.

Several previous studies using CT and anatomical dissection in ruminants species such as cows (Turgut et al. 2023), camels (Ben Khalifa et al. 2019; Kabkia et al. 2025), small ruminants (Masoudifard et al. 2022; Tohidifar et al. 2020) and buffaloes (Alsafy et al. 2013) have demonstrated the high degree of variability and specialization in paranasal sinus development. Despite interspecies differences, the frontal sinus remains the dominant component of the paranasal system across all these animals, forming multiple compartments that may expand into the cornual processes and even over the cranial vault. In Holstein cows, Turgut et al. (2023) similarly described a large, multi‐chambered frontal sinus that occupies much of the dorsal cranial cavity. Our observations confirm this pattern and emphasize the sinus's anatomical dominance and clinical importance.

In this study, CT images demonstrated that the chosen positioning protocol was well‐suited for cows, revealing symmetrical nasal structures. Transverse sections, acquired perpendicular to the hard palate, served as a consistent anatomical reference to allow for reproducible imaging across subjects. Dorsal reconstructions, obtained at right angles to the transverse plane, provided a broader overview of the nasal cavity architecture, aiding in the evaluation of potential pathological changes. Although a few sagittal views were included, their diagnostic value was limited due to the inability to simultaneously visualize both nasal passages, making side‐by‐side comparisons impractical. For these reasons, the transverse plane remains the most informative and reliable orientation for assessing nasal abnormalities.

The maxillary sinus, although less extensive than the frontal sinus, presents a noteworthy communication with the palatine sinus in the cow, forming a continuous cavity, a trait also noted in the buffalo (Alsafy et al. 2013). These interconnections between sinuses—some of which may not be clearly identifiable without cross‐sectional imaging—highlight the utility of CT over traditional techniques. Moreover, the conchal sinuses (particularly the dorsal conchal sinus) are consistently identified in the cow and other species such as the dromedary (Ben Khalifa et al. 2019; Kabkia et al. 2025), where they appear as relatively small but distinct compartments housed within the respective nasal conchae.

The sphenoid sinus in cattle, long considered underrepresented in the literature, has recently been studied in detail through sectional and 3D reconstructions (Turgut et al. 2024). This sinus lies ventral to the braincase, enclosed within the basisphenoid bone and tends to be symmetrically divided by a bony septum. Our findings confirm its medial position and relatively small volume in comparison to the frontal sinus, while also supporting the notion that this sinus becomes more pneumatized with age.

Ethmoidal cells, although not strictly classified as sinuses, play a significant role in nasal airflow and olfaction. They appear well developed in adult cows and exhibit a honeycomb structure within the ethmoid labyrinth. These were similarly observed in cross‐sectional studies in lemurs (Mogicato et al. 2012), suggesting phylogenetic conservation of their olfactory function, despite species‐specific differences in size and shape.

From a clinical standpoint, age‐related variations in sinus pneumatization and conchal development have diagnostic and surgical implications. In neonatal and juvenile cattle, underdeveloped sinuses may limit the spread of infections or complicate radiographic interpretation, as some sinuses may appear radiographically absent. Conversely, in adults, the extensive frontal sinus system increases the risk of sinus involvement in horn base injuries or dehorning complications. Understanding these ontogenic changes therefore assists clinicians in distinguishing normal developmental anatomy from pathological findings (Turgut et al. 2023).

However, in pathological conditions, the nasal cavities typically contain a mixture of components—such as bone, cartilage, mucosa and air—that exhibit varying physical densities. This heterogeneity often compromises the reliability of CT attenuation measurements. MRI, on the other hand, provides superior soft tissue contrast and can effectively distinguish nasal mucosa from surrounding soft tissues and fluid accumulations. Additional benefits of MRI include enhanced soft tissue delineation, higher spatial resolution for both normal and abnormal structures and the absence of ionizing radiation, making it a safer modality for repeated or prolonged imaging studies.

Regarding ontogeny, age‐related changes in the nasal cavities and paranasal sinuses are clearly evident. A recent study on New World monkeys (Smith et al. 2024) demonstrated age‐dependent changes, highlighting a gradual pneumatization of the paranasal sinuses. In calves, the frontal and maxillary sinuses are either absent or only partially pneumatized. As the animal matures, these structures progressively expand through pneumatization of diploic bone, often continuing well into adulthood. This expansion contributes to changes in skull shape and volume, which may impact surgical approaches or interpretations of CT images. The sphenoid sinus, for example, is rarely detectable in neonates but becomes visible and expands with age, as documented in our study and confirmed in buffalo (Alsafy et al. 2013) and Holstein cows (Turgut et al. 2023). The nasal conchae and ethmoidal cells, by contrast, show relatively minor morphological change after early development, indicating an early functional role likely linked to respiration and olfaction. These age‐related changes are essential to consider when interpreting CT images, especially in clinical contexts involving young animals. Misinterpretation of underdeveloped sinuses as pathological absence or aplasia can be avoided by knowledge of developmental anatomy. Furthermore, conditions such as sinusitis may manifest differently in immature animals due to these anatomical variations.

Finally, the contribution of CT to anatomical education should also be underlined. Three‐dimensional reconstructions allow for intuitive understanding of complex anatomical spaces, which is particularly valuable for veterinary students and practitioners dealing with surgical or diagnostic challenges in the head region (Chauhan et al. 2024; Erolin 2019). Moreover, CT allows in vivo, non‐invasive examination of the sinuses in animals of various ages, offering a window into their developmental dynamics.

However, several limitations must be acknowledged when interpreting the findings. A first important limitation concerns the small sample size and breed representation which restrict generalization and preclude statistical analysis. However, the aim was not to assess population‐level variation but to provide detailed descriptive and comparative imaging references across different ages, thereby complementing previous adult‐only anatomical studies. Future studies including larger populations and volumetric quantifications would strengthen these findings.

A second limitation concerns the non‐use of 3D volume rendering of our CT images. The added value of 3D volume rendering, as applied in studies on camels (Kabkia et al. 2025) and Holstein cows (Turgut et al. 2024), further facilitates surgical planning and anatomical education. Notably, in species with horned skulls, such as cattle, CT imaging is indispensable for evaluating horn base invasion by the frontal sinus, which can have clinical consequences in trauma or dehorning.

A last limitation relates to the use of CT imaging in vivo in cattle. The high cost of the equipment and procedure, together with the requirement for general anaesthesia, considerably restricts its routine application in clinical practice. General anaesthesia in adult cattle remains technically demanding and carries inherent risks due to the species’ body weight, ruminal physiology and susceptibility to regurgitation or tympany (Costea et al. 2024; Lin 2014). Consequently, CT examinations are generally reserved for animals of high economic or genetic value, such as breeding bulls or elite dairy cows, in cases where conventional diagnostic techniques are insufficient. The development of standing CT systems or advanced imaging protocols minimizing the need for general anaesthesia could, in the future, expand the clinical applicability of CT in large animals.

In conclusion, the nasal cavities and paranasal sinuses of the cow exhibit a complex and species‐specific morphology, characterized by large, interconnected cavities, particularly the frontal and maxillary sinuses. These structures undergo significant developmental changes with age, emphasizing the need for age‐specific anatomical references in diagnostic imaging. CT remains the imaging modality of choice for precise visualization of these regions, and its routine integration in both clinical and academic settings is strongly recommended.

## Author Contributions

**Charles Montel**: writing – original draft, methodology, visualization, software, data curation. **Florian Isler**: methodology, investigation. **Benjamin Cartiaux**: methodology, investigation. **Renaud Maillard**: review and editing. **Giovanni Mogicato**: conceptualization, writing – review and editing, investigation, data curation.

## Funding

The authors have nothing to report.

## Ethics Statement

The experimental protocol was approved by the ethical committee ‘Sciences et Santé Animale 115—Ecole Nationale Vétérinaire de Toulouse’ with the authorization number APAFIS# 21543‐201907191122830v2. All animals were euthanized for reasons unrelated to this study, following standard veterinary procedures using intravenous administration of pentobarbital sodium (Dolethal, Vetoquinol). Post‐mortem sample collection and experimental protocol were approved by the ethical committee.

## Consent

Informed owner consent was obtained.

## Conflicts of Interest

The authors declare no conflicts of interest.

## Data Availability

The data that support the findings of this study are available from the corresponding author upon reasonable request.
